# Thrombotic microangiopathy following haematopoietic stem cell transplant

**DOI:** 10.1007/s00467-017-3803-4

**Published:** 2017-10-09

**Authors:** Eleanor G. Seaby, Rodney D. Gilbert

**Affiliations:** 1 Human Genetics and Genomics Department, University of Southampton, Southampton, UK; 2 Southampton Children’s Hospital and Faculty of Medicine, University of Southampton, Tremona Road, Southampton, SO16 6YD UK

**Keywords:** Thrombotic microangiopathy, Hematopoietic stem cell transplant, Acute kidney injury, Complement, Neutrophil extracellular traps

## Abstract

Thrombotic microangiopathy is a potentially lethal complication of haematopoietic stem cell (bone marrow) transplantation. The pathophysiology is incompletely understood, although endothelial damage appears to be central. Platelet activation, neutrophil extracellular traps and complement activation appear to play key roles. Diagnosis may be difficult and universally accepted diagnostic criteria are not available. Treatment remains controversial. In some cases, withdrawal of calcineurin inhibitors is adequate. Rituximab and defibrotide also appear to have been used successfully. In severe cases, complement inhibitors such as eculizumab may play a valuable role. Further research is required to define the pathophysiology and determine both robust diagnostic criteria and the optimal treatment.

## Introduction

The thrombotic microangiopathies (TMAs) are a group of disorders defined by intravascular activation and sequestration of platelets, resulting in the accumulation of platelet- and fibrin-rich micro-thrombi that occlude and disrupt the microcirculation, fragmenting erythrocytes as they flow through the vessel lumen [1]. Disorders classified as TMAs include, but are not limited to: thrombotic thrombocytopaenic purpura (TTP); haemolytic uraemic syndrome (HUS); haemolysis, elevated liver enzymes and low platelets (HELLP) syndrome; and transplant-associated thrombotic microangiopathy (TA-TMA) [2]. Although these disorders share common features, with advances in genomics and histo-biology we are beginning to understand the aetiological differences that drive a final common pathway of endothelial damage that results in TMA and target organ damage [2, 3].

The first descriptions of TA-TMA were published in the early 1980s with observations of endothelial damage complicating bone marrow transplant in those receiving cyclosporine A for graft versus host disease (GVHD) prophylaxis [4, 5]. Since then, our understanding of what drives TA-TMA and exactly where it lies on the spectrum of TMA has been disputed and partially resolved. Features of TA-TMA include microangiopathic, Coombs-negative haemolytic anaemia and thrombocytopaenia, high serum lactate dehydrogenase (LDH) levels, decreased or undetectable haptoglobin levels, schistocytosis on peripheral blood smear, target organ damage and transfusion requirements. It is therefore understandable how the overlapping features of TA-TMA with most notably TTP and HUS have led to scientific debate about whether TA-TMA is merely a variant of TTP or HUS, or a completely distinct clinical entity [3, 6]. There is now convincing evidence that TA-TMA is distinct from TTP and may represent a type of HUS [2, 3], as demonstrated by its response to eculizumab therapy [7, 8]. Classical TTP results from a deficiency of a disintegrin and metalloprotease with a thrombospondin type 1 motif, member 13 (ADAMTS13) activity, leading to an accumulation of multimeric, prothrombotic von Willebrand factor (vWF) that drives intravascular thrombosis by the agglutination of circulating platelets under high shear stress [9]. In most patients with TA-TMA, ADAMTS13 activity is greater than 5 to 10%, metalloprotease activity is normal [3] and autoantibody inhibitors of vWF cleaving protease are undetectable, which is distinct from classical TTP. Furthermore, TTP is typically characterised by vWF in non-inflammatory, platelet-rich “white” thrombi [7], unlike the inflammatory, fibrin-rich “red” thrombi with C5b-9 deposition, caused by aberrant alternative pathway mechanisms of complement activation more commonly seen in HUS and TA-TMA [3, 7, 10, 11].

## Incidence

Reported incidence rates of TA-TMA vary widely, from 0.5 to 76% [10, 12–14], although more recently, sources aggregate the incidence between 12 and 35% [6, 15, 16]. Some of the challenge of estimating incidence lies in the extremely variable clinical presentation, ranging from asymptomatic anaemia, thrombocytopaenia or mild acute kidney injury to fulminating multisystem disease [6]. Different centres use different criteria for diagnosing TA-TMA (Table 1) and there may be difficulty distinguishing clinical features caused by other haematopoietic stem cell transplant (HSCT)-related complications, e.g. infection, GVHD and cyclosporine toxicity [14].

**Table 1:** Comparison of current diagnostic criteria for transplant-associated thrombotic microangiopathy (TA-TMA). NB: for Jodele’s criteria, if 1, 2, 3 are present, consider a diagnosis of TA-TMA and monitor very closely. Otherwise, 2 and 4 indicate features associated with poor outcome; therefore, consider therapeutic intervention [6, 12, 17, 18]

	Leukaemia Net International Working Group [17]	Blood and Marrow Transplant Clinical Trials Network [12]	Probably TMA [18]	Jodele’s proposed criteria [6]
Platelet count	<50,000/mm^3^ or <50% of baseline	N/A	<50,000/mm^3^ or <50% of baseline	<50,000/mm^3^ or <50% of baseline
Schistocytes	>4%	>2 per high power field	Present in peripheral blood or microangiopathy on tissue specimen	Present in peripheral blood or microangiopathy on tissue specimen
LDH	Increased	Increased	Increased	Increased
Haptoglobin	Decreased	N/A	De novo anaemia with haptoglobin below lower limit of normal	N/A
Transfusions	Increased	N/A	Anaemia requiring transfusion support	Hb below lower limit of normal for age or transfusion support
Direct Coombs test	N/A	Negative	Negative	N/A
Creatinine	N/A	2 x baseline	N/A	N/A
Coagulation studies	Normal	Normal	Normal	N/A
Terminal complement				Elevated plasma concentration of sC5b-9
Proteinuria	N/A	N/A	N/A	Random urinalysis proteinuria concentration of ≥30 mg/dL
Hypertension	N/A	N/A	N/A	3–18 years: BP at 95th percentile value for age, sex and height; > 18 years: BP ≥140/90 mmHg

* LDH* lactate dehydrogenase,* Hb* haemoglobin,* BP* blood pressure

## Aetiology and risk factors

Accurately discerning the aetiology of TA-TMA is hindered by the definition of TA-TMA. What is clear is that something intrinsic to HSCT provokes TA-TMA, and unpicking these triggers is important to understanding the pathogenesis of the disease. Several theories have been proposed to explain TA-TMA. Risk factors are likely to include intrinsic donor and recipient properties, in addition to extrinsic modifiers, such as conditioning regimens [19], viral infections, immunosuppressive therapies, especially combined sirolimus and cyclosporine regimens [20], and GVHD, all of which may damage endothelium and lead to a cascade of aberrant complement activation and TMA [7].

### Intrinsic factors

The most notable intrinsic factors associated with TA-TMA include female sex, advancing age and genetic predisposition [14, 21]. Although some reports have concluded that female gender is not a risk factor for TA-TMA [13, 22], other studies have found that females are statistically more likely to develop the disease than their male counterparts [14, 23]. The pathophysiology of this effect remains speculative; hypotheses include hormonal differences between men and women, potentially influenced by oral contraception use and pregnancy. Of note, HSCT from female donors is not an independent risk factor for TA-TMA, implicating the host environment as the source of increased risk [23].

With an increasing appreciation of how genetic predisposition underpins many diseases, focus has turned to the field of genomic medicine to help resolve disease risk and understand the biological mechanisms driving disease pathogenesis [24]. With overlapping features between aHUS and TA-TMA, genome sequencing has helped to close the gap between cited distinct entities and a reality of shared genetic aberrations in the alternative complement pathway. Jodele et al. [25], took an hypothesis-driven approach by assessing 17 genes in the alternative complement pathway, following the observation that complement activation (defined as elevated concentrations of plasma soluble C5b-9) at TA-TMA diagnosis predicts poor survival. They found that 65% of patients with TA-TMA had variants that increased alternative pathway complement activation in at least one of the 17 genes, whereas no known pathogenic variants were seen in the patients without TMA (*P* < 0.0001). Furthermore, variants in >3 genes were associated with higher mortality and were only seen in non-whites. This in part explains the racial disparity of TA-TMA incidence in this study and the previously described poorer outcome from HSCT in patients of African origin [26]. RNA sequencing has correlated gene variation (including variants predicted to be benign using in silico tools) with upregulation of complement activation [25]. These data indicate that dysregulated complement activation is central to the pathogenesis of TA-TMA and that genetic susceptibility plays a major role. Of course, this does not negate the importance of environmental stressors. For some, they may have less significant genetic susceptibility than others, yet experience stronger environmental stressors, and for others, they may be genetically vulnerable and develop TA-TMA with exposure to relatively fewer environmental stimuli. As genetic screening becomes easier, it may be possible in the future to identify those at highest risk of TMA before HSCT to allow closer follow-up and earlier therapy with complement-blocking drugs such as eculizumab [3, 27].

### External factors

Transplant-associated thrombotic microangiopathy occurs, on average, in 5–15% of patients after allogenic HSCT and in <1% after autologous HSCT. Pre-transplant conditioning, such as high-dose chemotherapy and total body irradiation are toxic to many cells and render the endothelium vulnerable [28]. Calcineurin inhibitors are commonly used in the immunosuppression regimen of HSCT and are directly toxic to endothelium. In a study on endothelial cells, both cyclosporine A and tacrolimus were proinflammatory; however, cyclosporine A exhibited significantly greater prothrombotic and proinflammatory effects [29]. Calcineurin inhibitors are frequently cited as a major risk factor for TA-TMA; indeed, their discontinuation has been shown to be effective in the treatment and sometimes reversal of the disease [22, 30]. A proposed mechanism has been that cyclosporine may suppress plasma levels of ADAMTS13 and limit its secretion. This theory is weakened by the >10% ADAMTS13 activity seen in TA-TMA. Furthermore, there is an incongruent association between TA-TMA disease severity and serum cyclosporine levels [7, 31]. However, this does not disprove the role of calcineurin inhibitors in modifying ADAMTS13 activity and being aetiologically significant in TA-TMA, but instead does not provide significant credence that (alone) these drugs are directly causal of TA-TMA. Sirolimus, a macrocyclic lactone antibiotic, is a therapeutic and prophylactic agent against GVHD. Unlike the calcineurin inhibitors, it has not been experimentally found to damage endothelium [29]. That said, its use in combination with calcineurin inhibitors potentiates the latter’s toxic effects, despite not conferring any independent risk for TA-TMA when used alone [32, 33]. Its concomitant use with cyclosporine has been shown to confer pro-necrotic and anti-angiogenic activities on endothelial cells. Some studies have shown a lower risk of GVHD and non-relapse mortality using a sirolimus/cyclosporine combination [20, 34], but a large, prospective study found no difference between a combination of tacrolimus and sirolimus compared with a combination of tacrolimus and methotrexate [35].

Much like the calcineurin inhibitors, acute GVHD has been associated with a four-fold higher incidence of TA-TMA [31], but it has been difficult to distinguish GVHD as an independent risk factor for TMA due to the confounding concomitant use of calcineurin inhibitors [6]. There has been further argument that TA-TMA is a variant of GVHD; however, the strongest piece of evidence to refute this claim stems from the observation that TA-TMA occurs following autologous HSCT, and that GVHD and TA-TMA can occur independently of one another [6, 7]. TMA may be misattributed to GVHD and thus initiate an increase in immunosuppression, but increased immunosuppression neither prevents nor treats TA-TMA [15]. In truth, GVHD and TA-TMA are not mutually exclusive and often overlap; both are associated with endothelial injury and they share many clinical features. Although cessation of immunosuppressants such as calcineurin inhibitors can be used to treat TA-TMA, their discontinuation may worsen GVHD and/or provoke GVHD recurrence. In patients with established GVHD and TMA, it is necessary to treat both diseases concurrently [15, 36].

Certain viruses, notably cytomegalovirus (CMV), adenovirus, parvovirus B19, BK virus and human herpes virus 6 (HHV-6) have been associated with the development of TA-TMA [37, 38]. It remains to be elucidated whether these particular viruses directly trigger TMA, or whether their presence is merely a reflection of overall disease severity post-HSCT [6]. That said, CMV and HHV-6 have been shown to directly damage endothelial cells and cause platelet aggregation [39, 40], and viral infections are a major activator of the alternative complement pathway [7].

## Pathophysiology

**Fig. 1:**
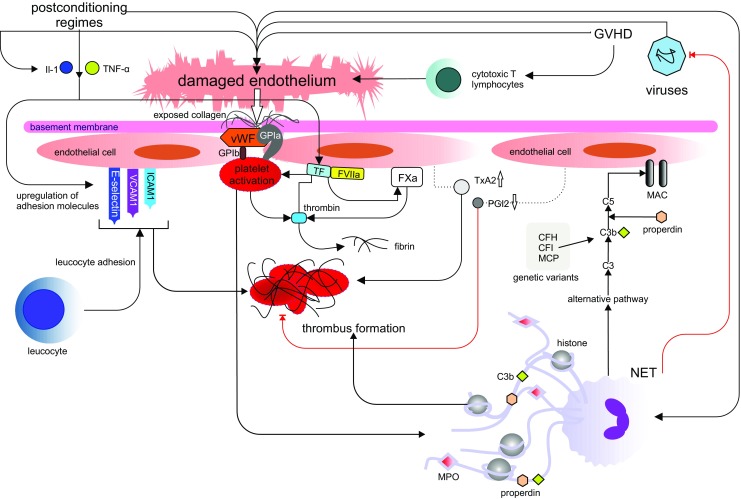
Endothelial injury pathways in thrombotic microangiopathies. Extrinsic factors from haematopoietic stem cell transplantation (HSCT), such as graft versus host disease (GVHD), post-conditioning regimens and viruses can all damage endothelium, either directly, or via chemotaxis and activation of donor cytotoxic T lymphocytes and upregulation of pro-inflammatory mediators such as interleukin 1 (IL-1) and tumour necrosis factor alpha (TNF-α). Furthermore, activated endothelium itself promotes IL-1 and TNF-α, perpetuating the inflammation and damage. Endothelial injury exposes collagen and negative charges, which binds von Willebrand factor (vWF), and GPIa, which facilitates platelet activation through the platelet glycoprotein GP1b. Concomitantly, exposed tissue factor (TF) forms a complex with activated factor VIIa and activating factor Xa, leading to the formation of thrombin. This, in turn, facilitates the further activation and aggregation of platelets, which, with the help of fibrin, forms a thrombus, capable of occluding vessels. TF, in addition to the aforementioned cytokines TNF-α and IL-1, upregulate soluble adhesion molecules, such as E-selectin, vascular cellular adhesion molecule 1 (VCAM-1) and intercellular adhesion molecule 1 (ICAM-1) to promote leukocyte adhesion and further promote thrombus formation. Increased levels of thromboxane A2 (TxA2) and reduced levels of suppressed production of prostaglandin I2 (PG12) observed in TA-TMA, fail to suppress platelet aggregation precipitated by cytokine-induced endothelial activation. Neutrophil extracellular traps (NETs), capable of killing viruses and fungi, are proposed to be a fundamental component of TA-TMA, promoting endothelial damage and thrombus formation. Furthermore, complement factor Bb and properdin are deposited on NETs, with consequent activation of the alternative complement pathway. Factors intrinsic to the host by means of genetic variants that alter the alternative complement pathway, can result in the unrestricted formation of C3 convertase on the endothelial cell surface, converting C3 to C3a and C3b, and ultimately resulting in injury to the endothelium through formation of the membrane attack complex (MAC) [6]. The pathophysiology of TA-TMA is a dynamic, contemporaneous process that involves many converging pathways. Inevitably, it is likely that an admixture of extrinsic and intrinsic factors, with relative impact, controls the process and severity of TA-TMA

There is limited consensus on the pathophysiology of TA-TMA, but there are a number of studies that provide sufficient data to allow the formulation of a hypothesis (Fig. 1). Most authors agree that endothelial damage is pathologically intrinsic to the disease process [10, 31, 32, 41]. This idea was born from studies that showed that microvascular endothelial cell apoptosis is induced after exposure to TTP and diarrhoea-negative HUS plasma, associated with the rapid induction of Fas CD95 [10, 42]. In addition, scanning electron microscopy has shown endothelial cell damage in TA-TMA, and soluble VCAM-1 and E-selectin levels are significantly elevated around the onset of TMA, giving further credence to the notion that endothelial damage might be a central event in a pathological process of procoagulation and platelet aggregation that results in the clinical features of TMA [43].

Plasma markers of endothelial injury are elevated in acute GVHD, and may be explained by a direct cytotoxic donor T lymphocyte attack on the endothelium. Activated endothelium promotes tumour necrosis factor alpha (TNF-α) and interleukin 1 (IL-1), which work synergistically to increase expression of plasminogen activator inhibitor 1 (PAI-1) and tissue factor, and TNF-α upregulates E-selectin, intercellular adhesion molecule 1 (ICAM-1) and platelet and endothelial adhesion molecule 1(PECAM-1 or CD31) to promote leukocyte adhesion [1]. Increased levels of IL-1, TNF-α and interferon gamma (IFN-γ) are commonly reported in the setting of acute GVHD and post-conditioning regimens [10, 32]. Suppressed production of PG1_2_ is also observed in TA-TMA; when depleted, it fails to adequately suppress platelet aggregation precipitated by cytokine-induced endothelial activation [10, 41]. This is perhaps augmented by cyclosporine’s effect on the increasing production of thromboxane A2 and thus inhibits the production of prostacyclin (PGI_2_) [1].

The microvascular endothelium is a complex tissue that synthesises a multitude of factors governing coagulation and fibrinolysis. Such substances include tissue factor, ICAM-1, vWF, thrombomodulin, PAI-1, PG1_2_ and nitric oxide. Variations in the abundance of these factors is shown to be associated with TMA [32, 40], either caused by intrinsic factors that render the endothelium vulnerable to activation, or by directly toxic insults as associated with HSCT. The sequence of events following endothelial damage includes intimal swelling and fibrinoid necrosis of the microvascular wall, leading to platelet aggregation, partly occluding the vessel lumen. Erythrocytes are mechanically sheared by the fibrin-rich micro-thrombi, resulting in the clinical manifestations of Coombs-negative microangiopathic haemolysis, raised bilirubin, raised LDH and reduced haptoglobin.

In addition to their traditional role in the innate immunity of phagocytosis and intracellular killing of pathogens, certain neutrophils, especially low-density neutrophils, can extrude their DNA and associated peptides such as histones and myeloperoxidase to form reticular structures known as neutrophil extracellular traps (NETs) that can kill a wide variety of pathogens from viruses to fungi [44]. Although the factors leading to NETosis (the process of NET formation) are incompletely understood, we do know that NETs can damage endothelial cells [45]. Patients with a variety of TMAs have elevated levels of NETs in the plasma and remission is associated with a return to normal levels [46]. Damaged endothelium induces NET formation, which in turn drives further endothelial damage [47]. Arai et al. [16] showed an association between NET levels pre- and post-HSCT, with higher levels correlated with the development of TA-TMA and directly visualised NETs in the renal glomeruli. Furthermore, complement factor Bb and properdin are deposited on NETS with consequent activation of the alternative complement pathway, both on the NETs and in the serum [48].

Complement serves to provide host immunosurveillance, aiding the clearance of foreign and apoptotic cells. Under physiological conditions, circulating complement factor H (CFH) works together with membrane regulators by binding to host–cell surfaces and inhibiting complement attack by the alternative complement pathway [49]. In aHUS, regulatory proteins of the alternative complement pathway are frequently defective. This is either genetically determined or acquired through the presence of complement factor H or factor I autoantibodies, and results in unrestricted formation of C3 convertase on the endothelial cell surface, converting C3 to C3a and C3b. In turn, this promotes the formation of C5 convertase, resulting in the formation of the membrane attack complex and the anaphylatoxin C5a, which cause injury to the endothelium [6]. Interestingly, the complement staining pattern of TA-TMA resembles that of aHUS, with C4d deposition localised to the glomeruli and arterioles [17], offering an historical record of complement activation, although it should be noted that C4d staining is also seen in a small number of patients without evidence of TA-TMA [8]. This suggests that TA-TMA might represent aHUS in the setting of HSCT and this theory is certainly supported by the enrichment of variants in complement genes seen in TA-TMA versus HSCT without TMA [25]. These findings suggest that a genetically weakened complement regulatory system is vulnerable to HSCT-related factors that can further activate the complement system and directly damage microvascular endothelium. The role of complement is further supported by the observation that elevated plasma concentrations of the terminal complement complex, C5b-9, correlate with severe disease and poor outcome, in addition to reports of the beneficial effect of eculizumab treatment in patients with TA-TMA [8].

It can therefore be hypothesised that in certain patients, factors such as genetic predisposition, underlying disease, conditioning regimen, GVHD or infection might lead to exuberant NETosis and/or reduced clearance of NETs. This leads to endothelial damage and complement activation. In patients with genetic variants reducing inhibition of alternative pathway complement activation, this can lead to life-threatening TMA.

### How TA-TMA specifically affects the kidney

Renal biopsy specimens of TA-TMA patients show mesangiolysis and loss of endothelial cells, with subendothelial expansion and luminal occlusion by fragmented erythrocytes, fibrin deposition and necrotic products [31]. C4d deposition in the glomerular and peritubular capillaries is common to TA-TMA and aHUS [17]. Inflammatory infiltrates, including natural killer cells, CD3+ and CD8+ T cells and cytotoxic T cells, have been found within the kidney glomeruli, interstitium and tubules [31]. The resulting clinical presentation includes proteinuria, hypertension and decreased glomerular filtration rate (GFR), yet these may also be observed in transplant recipients without TA-TMA, i.e. those exposed to nephrotoxic and hypertension-inducing medicines and certain bacterial and viral infections [6]. That said, renal TA-TMA should be suspected when HSCT recipients require more antihypertensive therapy than would be expected for GVHD prophylaxis or treatment, and should especially be considered in patients requiring more than two antihypertensive therapies [6] or with nephrotic-range proteinuria [11].

### Extra-renal features

Transplant-associated thrombotic microangiopathy is not merely a renal disease; vascular involvement of extra-renal organs such as the heart, lungs and gastrointestinal tract have all been reported in TA-TMA and multi-organ involvement confers a higher morbidity and mortality [15, 18].

#### Heart and lungs

The incidence of cardiac and pulmonary complications post-HSCT has been reported in as many as 30% of cases, and includes pericardial effusion (PEF), elevated right ventricular (RV) pressure and reduced left ventricular function. Raised right-sided pressures have been significantly associated with TA-TMA; indeed, in a single-centre prospective study, Dandoy et al. found that nearly 70% of patients with elevated RV pressures, as detected by echocardiogram on day 7 post-HSCT, were subsequently diagnosed with TA-TMA [50]. Furthermore, all patients with both PEF and raised RV pressures had concomitant TA-TMA, potentially representing a polyserositis [18]. Histological evidence for pulmonary arteriolar microangiopathy includes injured endothelium, microthrombosis and schistocyte extravasation into the pulmonary interstitium [6]. As a result, pulmonary artery pressures rise and RV failure may ensue. These authors propose that elevated RV pressures may indicate early pulmonary vascular injury that can precede haematologically detectable TA-TMA by current diagnostic criteria. The importance of identifying pulmonary involvement early is imperative, as post-transplant survival at 1 year is statistically worse in those with cardiac and pulmonary involvement [50]. Left untreated, pulmonary hypertension in the presence of TA-TMA has a high mortality rate [6].

#### Gastrointestinal tract

Severe diarrhoea, abdominal pain and bleeding are common and serious adverse events post-HSCT, frequently blamed on infection or gut GVHD. However, in many cases, these symptoms, most notably abdominal pain and bleeding, are refractory to increased immunosuppression, implicating the gastrointestinal (GI) tract as another target for TMA [51]. As GVHD and TA-TMA can occur concurrently, increased immunosuppression tends to treat the voluminous diarrhoea caused by GVHD, whilst leaving the bowel susceptible to ischaemia by TMA. In fact, biopsies from colonoscopy studies of clinically diagnosed acute gut GVHD have been reported to show histological evidence of TA-TMA in >90% of patients, with as few as 30% of patients actually having histological evidence of GVHD [52]. Therefore, abdominal pain and rectal bleeding should raise the possibility of TA-TMA. Histologically, gut TMA presents as endothelial cell separation from the vascular wall, aggregation of intraluminal fibrin, microthrombi and schistocytes, with denudation of mucosa and loss of glands [51]. Differentiating gut GVHD from gut TMA is difficult, not least because they share some histological features and can occur concomitantly [6, 51]. The histological unpicking of GI disease aetiology is essential, as treatment regimens are antipodal; calcineurin inhibitors treat GVHD, whilst their discontinuation is one of the mainstay treatments for TA-TMA [6].

#### Central nervous system

Central nervous system (CNS) involvement in TA-TMA is not fully understood. Patients with TA-TMA often have neurological symptoms, including headache, seizures, visual disturbances, confusion and hallucinations, but many of these symptoms may be attributed to metabolic disturbances or hypertension complicating TA-TMA [6]. If hypertension is uncontrolled, there is a risk of posterior reversible encephalopathy syndrome (PRES), an acute encephalopathy syndrome characterised by seizures, altered mental status and visual disturbances [53]. PRES, like TMA, is a disease of endothelial injury. It occurs because of abrupt changes in blood pressure or from direct cytotoxicity of the blood–brain barrier. Radiological findings include bilateral vasogenic oedema concentrated in the subcortical parieto-occipital regions. As the name states, PRES is considered reversible; however, in some cases cerebral haemorrhage ensues, which can lead to significant morbidity or mortality from brain infarction and brainstem compression respectively [54].

## Diagnosis

Currently, there are no universally accepted diagnostic criteria for TA-TMA; nevertheless, attempts have been made to create a framework for diagnosis, and broadly speaking, thrombocytopaenia, schistocytosis, increased LDH, decreased haptoglobin, a negative direct Coombs test with normal coagulation studies, and exclusion of TTP by >10% ADAMTS13 levels are accepted clinical features that warrant diagnosis (Table 2). The difficulty in creating diagnostic criteria is that TA-TMA shares many clinical features with sequelae of HSCT, limiting the specificity of any one feature. Taking each clinical criterion in turn, it can be argued that alternative pathological processes are responsible. For example, thrombocytopaenia may occur as a result of delayed megakaryocyte engraftment, infection, GVHD and myelosuppressive therapies [1]. Schistocytosis is not uncommon in transplant patients, and may be attributed to infection, underlying haematological malignancies or disseminated intravascular coagulation [7]. Furthermore, the assessment for schistocytes is a somewhat subjective test requiring haematological screening of peripheral blood smears, and reporting may vary among and within laboratories [55]. Haptoglobin and LDH levels are again non-specific findings and institutional laboratory values vary.

**Table 2:** List of genes related to transplant-associated thrombotic microangiopathy (TA-TMA)

TA-TMA-related genes
*CFH*
*CFHR1*
*CFHR3*
*CFHR4*
*CFHR5*
*CD55*
*CD59*
*CD46*
*CFI*
*CFB*
*CFP*
*C5*
*ADAMTS13*
*CFD*
*C3*
*C4BPA*
*THBD*

To complicate matters further, the presentation of TA-TMA is extremely variable. Disease severity can range from asymptomatic low-grade disease confined to the kidneys, to fulminant, systemic disease involving the lungs, heart, gut and brain [1, 6]. That said, there should be a high index of suspicion for TA-TMA with multi-organ involvement post-HSCT. Of course, detecting TA-TMA before there is multi-organ failure is of paramount importance, and arguably the current diagnostic criteria are more sensitive to established disease, i.e. schistocytes on peripheral blood smear may represent a late sign of vascular injury [50], and decreased haptoglobin levels and doubling of serum creatinine present 2 and 4 weeks respectively after diagnosis [15]. Consequently, research has focused on finding early markers that may indicate TA-TMA. Jodele et al. [6] suggest that elevated LDH, proteinuria and hypertension might be early markers of disease, and might precede diagnosis by 10–14 days [15]. Statistically significantly more patients with TA-TMA versus without developed high random urine protein:creatinine ratios and required more anti-hypertensive therapy [55]. Elevated sC5b-9 levels provide another early marker of TA-TMA that often precedes haematological markers; however, it is not a readily available clinical test. Other suggested early diagnostic markers include elevated RV pressures and post-transplant pericardial effusion. Dandoy et al. suggest that echocardiographic detection of pulmonary hypertension might be a good and sensitive screening tool [50].

Once TA-TMA is established, patients can be tested for complement factor H autoantibodies and mutations in genes associated with the complement pathway, but diagnosis should not depend on the demonstration of deleterious genetic variants. When testing DNA, it is important to ensure that the DNA is truly representative of the recipient. After stem cell transplantation and engraftment, DNA extracted from the recipient’s blood reflects the donor DNA; therefore, DNA should be obtained either from a stored sample before transplant or from a tissue site other than blood. Histology can support the diagnosis, but is not possible for every individual, particularly in the setting of thrombocytopaenia.

## Treatment

Therapeutic approaches to TA-TMA include supportive therapy and targeted disease therapy. First-line therapy should include the minimisation of factors that may precipitate TA-TMA. Examples include withdrawal of calcineurin inhibitors and/or co-therapy with sirolimus, although this should be measured against the risk of precipitating GVHD, which is itself an independent risk factor for TA-TMA. Adequate antibiotic therapy is essential to minimise potentially fatal infections in immunocompromised patients, as is aggressive antihypertensive therapy and renal replacement therapy [6, 21].

### Plasma exchange

The therapeutic use of plasma exchange in TA-TMA lacks convincing evidence. Although effective in TTP, its use in TA-TMA yields limited benefit, with response rates below 50% [21]. In TTP, plasma exchange is effective because it removes autoantibodies that deplete ADAMTS13 and replenishes supplies. In TA-TMA, and indeed aHUS, the response to plasma exchange is limited, although its efficacy is possibly influenced by confounding factors such as the time of disease presentation post-HSCT [56], GVHD [57] and timing of clinical intervention [58]. Moreover, clinicians must be cautious of pseudo-responses, reflected by improvements in haematological parameters such as platelet count and haptoglobin levels; fresh frozen plasma is rich in complement regulatory proteins that can transiently appear as if the disease process is relenting, when, in fact, despite response rates between 37 and 55%, the pathological process continues and mortality rates remain high at 80% [1, 14, 58, 59]. Moreover, plasma exchange harbours adverse effects, such as alloimmunisation, thrombosis, pneumothorax, pericardial tamponade, serum sickness, vascular catheter infections, and infusion reactions, such as anaphylaxis and transfusion-related acute lung injury [7].

### Immunotherapies and anti-thrombotic therapies

The anti-CD20 monoclonal antibody therapy, rituximab, has proven efficacy in TTP with ADAMTS13 activity levels <10% [7] and in the prevention of aHUS with CFH autoantibodies after kidney transplantation [15]. The empirical therapeutic use of rituximab in TA-TMA has been largely positive, achieving remission in patients without suppressed ADAMTS13 activity, most likely by acting as an immunomodulatory agent [60]. Of course, the possibility of reporting bias cannot be excluded, as cases with poor outcomes may be reported less commonly. Its mechanism of action in TA-TMA is not fully understood, although it is hypothesised that it may act by depleting CD20+ B cell precursors, thus attenuating T-cell activation and cytokine release [60]. Defibrotide is a polydeoxyribonucleotide that has shown promise as a potential TA-TMA therapy through its action on platelet aggregation and endothelial protection against calcineurin inhibitors, without a significant effect on systemic coagulation [29, 55, 61]. Corti showed defibrotide to be beneficial in approximately 50% of patients post-HSCT [61]. That said, the studies on defibrotide lack substantial numbers and data to ratify some of the claims made on its efficacy.

### Complement targeted therapies

Perhaps the most promising therapy for TA-TMA is eculizumab, a terminal complement inhibitor that is highly effective in treating aHUS. With increasing evidence for the role of dysregulated activation of the alternative complement pathway underpinning endothelial damage that drives TMA, focus has turned to drugs that act to inhibit the complement system.

Eculizumab is a recombinant, fully humanised, monoclonal antibody that binds complement C5 and blocks production of proinflammatory C5a and the membrane attack complex C5b-9 [27]. Its use in aHUS has proved life-saving for many patients. With considerable overlap between aHUS and TA-TMA both genetically and histologically, eculizumab has been used for both the prevention and treatment of TA-TMA. However, its use and efficacy are not interchangeable and the drug exhibits varying pharmacokinetics and pharmacodynamics. Thus far, the outcomes have been favourable. Indeed, Jodele et al. showed improved survival in a small cohort of patients with high-risk TA-TMA treated with eculizumab compared with alternative treatments. One-year overall survival was 62% vs 9% (*P* = 0.0007), although in some patients it has not been possible to achieve therapeutic plasma concentrations. For the best results, eculizumab administration in TA-TMA requires a personalised drug-dosage regimen that is titrated against a variable drug clearance, which is determined by disease severity [62]. Importantly, available evidence suggests that eculizumab therapy might not need to be continued long term and can safely be stopped after TA-TMA resolution, offering an effective and curative treatment. It is also important to give TA-TMA patients on eculizumab prophylactic antibiotics to prevent meningococcal disease. Usually, a polyvalent meningococcal vaccine is administered 2 weeks before the first dose of eculizumab, as complement blockade renders the immune system vulnerable to meningococci. As post-transplant recipients are severely immunocompromised, they are unable to mount a vaccine response until their adaptive immune system is reconstituted. Reassuringly, evidence has shown that with adequate antibiotic prophylaxis, there is no increased risk of meningococcal disease in non-vaccinated eculizumab recipients [63].

## Outcome/prognosis

Transplant-associated thrombotic microangiopathy causes significant morbidity and mortality, with reported death rates as high as 75% within 3 months of diagnosis [12]. Research has focused on finding early prognostic factors to aid faster diagnosis, quicker intervention and predict those at highest risk of an unfavourable disease trajectory.

Factors associated with poor outcomes include proteinuria (>30 mg/dL) and raised plasma levels of sC5b-9 at diagnosis [11]. Jodele et al. showed that patients with elevated plasma concentrations of the terminal complement complex sC5b-9 and proteinuria at diagnosis had an 84% non-relapse mortality rate 1 year post-HSCT; all patients without proteinuria and elevated sC5b-9 survived [6]. Pulmonary hypertension is another poor prognostic factor and mortality rates reach 80% [55]. However, with evidence that pulmonary hypertension may precede haematological diagnostic parameters, these rates may decrease if prompt treatment is administered.

## Conclusion

Transplant-associated thrombotic microangiopathy is a multi-faceted disease process underpinned by host factors and external stressors. Now understood as a separate entity to TTP, but as a tissue microangiopathy that closely mimics aHUS, we are beginning to gain a better understanding of the pathogenesis of this devastating disease. The discovery of NETs and their role in endothelial damage, thrombus formation and complement activation may prove to be an integral component of TMA pathophysiology. Despite efforts to improve outcomes over recent years with biological drugs such as eculizumab, TA-TMA remains a potentially fatal complication of HSCT. Identifying those at greatest risk of TA-TMA and recognising the early warning signs of disease will inevitably improve survival and outcomes.

## Key summary points

1. Transplant-associated thrombotic microangiopathy is a frequent complication of stem cell transplants, sharing clinical features with GVHD. It is important to distinguish between GVHD and TA-TMA and where there is an overlap, it is essential to treat both diseases.
2. Dysregulated complement activation plays a central role in the pathogenesis of TA-TMA. Multiple factors, both genetic and environmental, may contribute to abnormal complement activation.
3. Transplant-associated thrombotic microangiopathy strongly overlaps with aHUS, sharing many genetic disease risk alleles. Eculizumab therapy has proved successful in the treatment of both aHUS and TA-TMA, and in many cases provides a definitive cure. For TA-TMA specifically, patient-specific drug dose regimens are required to ensure optimal pharmacokinetics in a disease that exhibits varying drug clearance in changing states of disease activity.
4. Endothelial damage is intrinsic to the pathogenesis of TA-TMA. There is emerging evidence that neutrophil extracellular traps (NETs) may play a crucial role in the pathophysiology of endothelial damage and complement activation.

## Questions (answers are provided following the reference list)

- 1. Which of the following statements is false?

- Transplant-associated thrombotic microangiopathy is more common following autologous haematopoietic stem cell transplantation.
- Calcineurin inhibitor treatment may predispose patients to TA-TMA.
- Some patients may be genetically predisposed to developing TA-TMA.
- Conditioning regimens may predispose to TMA.
- Pulmonary hypertension 1 week after transplantation may indicate a high risk of later development of TA-TMA.

- 2. Which two of the following statements are false?

- Low platelet count does not confirm a diagnosis of TA-TMA.
- Diarrhoea in a recipient of a haematopoietic stem cell transplant can be caused by infection or graft versus host disease, but not by TA-TMA.
- Patients with TA-TMA may have multisystem disease, including cardiac and CNS involvement.
- Diagnosis of TA-TMA can be aided by biopsy of affected tissues.
- There are no internationally accepted criteria for establishing a diagnosis of TA-TMA.

- 3. Which of the following is NOT an appropriate therapeutic choice in a patient with TA-TMA:

- Treatment with eculizumab
- Treatment with rituximab
- Treatment with defibrotide
- Treatment with cyclosporine and sirolimus

- 4. Which two of the following statements are true?

- Untreated, TA-TMA can be a devastating disease with a very high mortality rate.
- Plasma exchange is useful, as it replenishes ADAMTS13 levels.
- If eculizumab treatment is started, it needs to be continued throughout life.
- Patients with TA-TMA almost all have very low plasma concentrations of complement C3.
- The presence of hypertension and heavy proteinuria together strongly suggests a diagnosis of TA-TMA.

- 5. Which of the following statements is false?

- Endothelial damage caused by conditioning regimens may contribute to the pathogenesis of TA-TMA.
- Complement activation, especially the alternative pathway, appears to play an important role in the pathogenesis of TA-TMA.
- NETosis is an attempt by the body to combat TA-TMA and therapies that promote it are likely to be effective at treating TA-TMA.
- Platelet activation probably plays an important role in the pathogenesis of TA-TMA.
- Neutrophil extracellular traps can activate complement and damage endothelial cells.
